# Targeted ^19^F MRI for molecular imaging of the immune response and its theranostics

**DOI:** 10.1093/immadv/ltaf035

**Published:** 2025-12-01

**Authors:** Pascal Bouvain, Sebastian Temme, Ulrich Flögel

**Affiliations:** Experimental Cardiovascular Imaging, Molecular Cardiology, Heinrich Heine University, Düsseldorf 40225, Germany; Department of Anesthesiology, University Hospital Düsseldorf, Düsseldorf 40225, Germany; Cardiovascular Research Institute Düsseldorf (CARID), Heinrich Heine University, Düsseldorf, Germany; Experimental Cardiovascular Imaging, Molecular Cardiology, Heinrich Heine University, Düsseldorf 40225, Germany; Cardiovascular Research Institute Düsseldorf (CARID), Heinrich Heine University, Düsseldorf, Germany

**Keywords:** targeted nanoparticles, theranostics, neutrophils, fluorine, MRI, inflammation

## Abstract

The *in vivo* visualization of inflammatory processes offers not only the possibility of localizing disease but also of monitoring its progression over time. Among available imaging modalities, combined ^1^H/^19^F MRI has emerged as a powerful technique, as it enables the detection of inflammation with minimal background signal. Beyond diagnosis, however, there is increasing interest in using this platform to initiate targeted therapeutic interventions. The integration of these two components—diagnosis and therapy—is commonly referred to as theranostics. In this review, we provide an overview of the potential of fluorine-based MRI and highlight a range of targeted theranostic applications across different disease models. Particular emphasis is placed on recent strategies to manipulate neutrophils during acute colitis, which have demonstrated significant improvements in disease outcome.

## Introduction

### The immune system

Pathogenic challenges are daily threats for every organism. In normal circumstances, it is difficult for microbes to pass the anatomical and physiological barriers of the human body. However, pathogenic bacteria and viruses can infect the tissue *via* the epithelial lining of the mucosal tissue and induce an inflammation. Inflammatory environments are governed by different immune cells infiltrating the inflammatory hot spot as well as the release of pro-resolving factors to challenge invading pathogens leading from a pro- to an anti-inflammatory state while recognizing the host organism and limiting damage to itself. In the recent years, it has become more and more clear that the coordination of these complex immune processes is of huge medical relevance. On the one hand, a proper orchestration of the inflammatory response is essential to fight against pathogenic microorganisms, on the other hand, the immune system is also crucially involved in the development and progression of multiple diseases which are not caused by pathogens like myocardial infarction [1], atherosclerosis [2], or even schizophrenia [3].

### Innate and adaptive immunity

**Figure 1.**
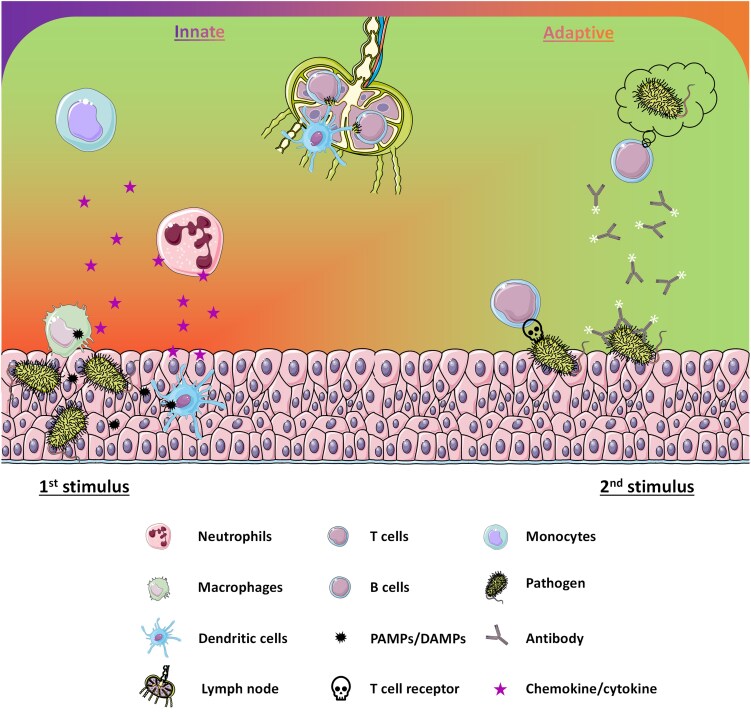
The immune system is divided into the innate and adaptive immune response. In response to bacterial infections, pathogen-associated molecular patterns (PAMPs) are released that activate surrounding immune cells like tissue macrophages or dendritic cells. These cells secrete cytokines/chemokines to attract other immune cells such as neutrophils and later also monocytes. This first innate response is usually sufficient to control most of the invading pathogens. In parallel, dendritic cells become activated and migrate into the draining lymph nodes to present antigenic peptides or lipids of the pathogens to T and B cells. In the event of further infection, the adaptive immune system is directly involved and B as well as T cells are able to remember the pathogens and either secrete antibodies or eliminate cells infected by these pathogens.

Over millions of years, the immune system has repeatedly adapted and developed to changing circumstances. The immune system in vertebrates is roughly divided into two complementary arms: the innate and the adaptive immune systems. The innate immune system serves as the body’s first line of defense against invading pathogens. It consists of anatomical and physiological barriers, antimicrobial enzymes like lysozymes in saliva and mucus and a diverse population of immune cells. In addition, the skin and gut microbiota contribute to host protection by outcompeting pathogenic microbes. The critical role of innate immunity becomes evident in individuals with immunodeficiencies, who are more susceptible to severe and life-threatening infections [4]. Upon tissue injury or pathogen invasion, danger-associated molecular patterns (DAMPs) and pathogen-associated molecular patterns (PAMPs) are released [5]. These molecules are recognized by innate immune cells such as macrophages and dendritic cells, which initiate an inflammatory response through the secretion of cytokines and chemokines. This process activates endothelial cells and promotes the recruitment of immune cells from the bloodstream into the damaged tissue. Neutrophils are one of the first responders and play a central role in pathogen clearance through mechanisms such as phagocytosis, the release of reactive oxygen species and NETosis. Monocytes, which arrive shortly after neutrophils, differentiate into macrophages or dendritic cells at the site of inflammation and further coordinate the immune response (Fig. 1, left). If the innate response is insufficient or if the body encounters the same pathogen again, the adaptive immune system is activated. This system features highly specific T and B lymphocytes capable of recognizing antigens presented by antigen-presenting cells. Dendritic cells play a pivotal role in this process by migrating to lymph nodes, where they present processed antigens *via* MHC (major histocompatibility complex) molecules to CD4^+^ helper T cells and CD8^+^ cytotoxic T cells. The latter eliminate infected cells, while helper T cells support B cell activation and antibody production, as well as macrophage function at the inflammation site. A hallmark of the adaptive immune system is the development of immunological memory, allowing for faster and more effective responses upon re-exposure to the same pathogen (Fig. 1, right). This feature is the foundation of vaccination, which aims to induce long-lasting protection against infectious agents. Together, the innate and adaptive immune systems form a highly coordinated and dynamic network essential for maintaining immune surveillance, eliminating pathogens, and resolving inflammation.

## Imaging of inflammation

As outlined above, the innate and adaptive immune response is a highly complex system that is constantly changing and interacting with the environment in order to suppress the onset of disease. It is therefore obvious that the visualization of immune cells *in vivo* covers an important area of clinical research in order to be able to make a timely statement about the current status and also the course of a disease. This urgency is not only the case for acute diseases caused by pathogens but also in in chronic states such as cardiovascular diseases, neurodegenerative disorders or even cancer. For this reason, it is important to be able to visualize inflammatory processes *in vivo*. This not only enables the exact localization of the site of inflammation but also allows for long-term studies to monitor the further development of a disease. Various imaging modalities are available for this purpose, such as positron emission tomography (PET), single-photon emission computed tomography (SPECT), or magnetic resonance imaging (MRI), which enable long-term examination of a disease and have a sufficient penetration depth. However, beside PET and SPECT which both rely on radioactive nuclides, MRI provides superior soft tissue contrast and has proven to be a very versatile tool for tracking specific inflammatory processes *in vivo* by combining the conventional proton imaging (^1^H) with different contrast agents like gadolinium (Gd), manganese (Mn), superparamagnetic iron oxide nanoparticles (IO-NPs), or fluorine (^19^F).

### Conventional contrast agents for ^1^H MRI

Gadolinium (Gd^3+^) is a highly paramagnetic ion; the presence of seven unpaired electrons confers a substantial magnetic moment, resulting in a strong T1-relaxation enhancement of adjacent water protons. Gd-based contrast agents (GBCAs) are the most widely used MR-CAs with ∼63 million doses in 2023 [6]. However, free Gd^3+^ is highly toxic and is associated with the development of nephrogenic fibrosis in patients with impaired kidney function [7]. More recently, it has been found that GBCAs accumulate in the brain of patients with normal kidney function who received repetitive Gd-based MRI examinations which has again questioned the safety of GBCAs [8]. Nevertheless, the overall safety-profile of GBCAs is good with only few adverse events [8]. GBCAs are mostly used for diagnostics of the brain, the cardiovascular system, the abdomen, and the musculoskeletal system [6].

Manganese (Mn^2+^) is another paramagnetic MR-contrast agent with five unpaired electrons. Similar as GBCAs, accumulation of Mn^2+^ ions induce bright signals in T1-weighted images but also darkens T2-weighted MR-images and therefore has also been used as dual-mode agent [9]. For contrast enhanced MRI, Mn^2+^ can be used as salt (MnCl_2_). Soluble Mn^2+^ enters the cells *via* Ca^2+^ channels and has been used for visualization of viable myocardium after myocardial infarction in mice [10].

Iron oxide nanoparticles (IO-NPs) exhibit strong superparamagnetic behavior when placed in an external magnetic field. This effect disturbs the local magnetic field and enhances the dephasing of the spins of the surrounding water molecules which results in a strong decay of the T2 signal and leads to the darkening of the anatomical ^1^H MR image [11]. Some IO-NPs are avidly taken up by phagocytic immune cells and have been used in preclinical, but also clinical studies to visualize the accumulation of monocytes/macrophages in inflammatory lesions [12]. One important advantage of IO-NPs is the very high sensitivity which has enabled even single-cell tracking of monocytes in the brain [13].

Despite excellent sensitivity, all these approaches have the disadvantage that local deposition of those CAs creates hypo/hyperintense regions in affected tissues with the entire anatomy of the investigated object as background signal, which makes an unambiguous identification *in vivo* difficult or even impossible.

### Detection of inflammation by combined ^1^H and ^19^F MRI

**Figure 2.**
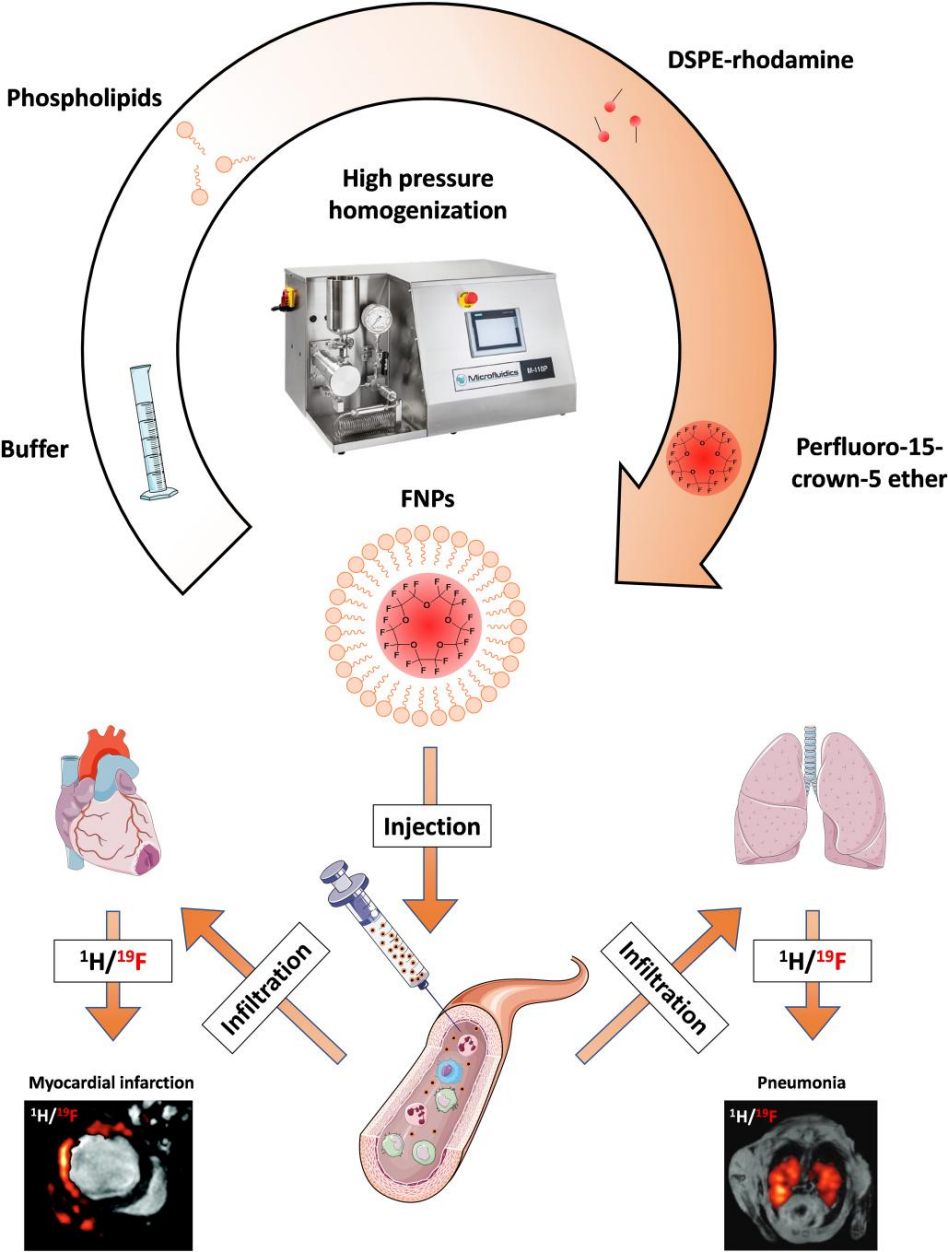
FNPs are prepared by mixing buffer with lipids, fluorescent-labeled lipids for flow cytometry or microscopy and the perfluoro-15-crown-5 ether followed by high pressure homogenization at 1000 bar. Upon injection into the blood stream, FNPs are taken up by different immune cells from the blood, e.g. monocytes and neutrophils. In case of an inflammation these labeled cells infiltrate into the inflammatory hot spot and can be visualized *via* combined ^1^H/^19^F MRI. Adapted from Flögel *et al.* 2008 (left; myocardial infarction) [14] and Ebner *et al.* 2010 (right; pneumonia) [15].

As an alternative, ^19^F MRI has emerged in the recent years as a powerful tool for imaging of inflammatory processes. A major advantage of this technique is that fluorine is virtually absent from the human body, meaning that externally administered fluorine-containing contrast agents can be clearly detected without any significant background interference. Moreover, the resulting fluorine signal can be easily merged with anatomical ^1^H MR images, enabling its precise localization. However, a limitation of fluorinated compounds with a high payload of ^19^F nuclei is their hydrophobic nature, making them insoluble in water and most other biological fluids (e.g. blood). To overcome this, they must be formulated as nanoemulsions through high-pressure homogenization to form fluorine-based nanoparticles (FNPs) (Fig. 2, top). In a groundbreaking paper Ahrens *et al*. demonstrated that dendritic cells can be labeled *ex vivo* with such FNPs and subsequently tracked *in vivo* following intramuscular injection [16]. A few years later, our working group went one step further and applied the FNPs directly *in vivo*. After intravenous injection, these nanoemulsions are taken up by phagocytic immune cells in the bloodstream, particularly monocytes and neutrophils. In the presence of inflammation, these labeled immune cells migrate to the inflammatory foci, where they can be visualized using combined ¹H/¹⁹F MRI. This approach allows for the tracking of immune cell infiltration across a wide range of diseases, including myocardial infarction [14], graft rejection [17], or pulmonary inflammation [15] (Fig. 2, bottom). However, some studies have also shown that, under certain conditions, other cell types—such as neutrophils [18] or even specific stem cell populations in the heart [19]—can also be labeled upon intravenous administration of FNPs, depending on the time point of injection. Beyond this passive labeling by phagocytosis, cells and other epitopes can also be actively targeted *via* specific ligands that have been coupled to the FNPs.

### Active targeting of FNPs

**Figure 3.**
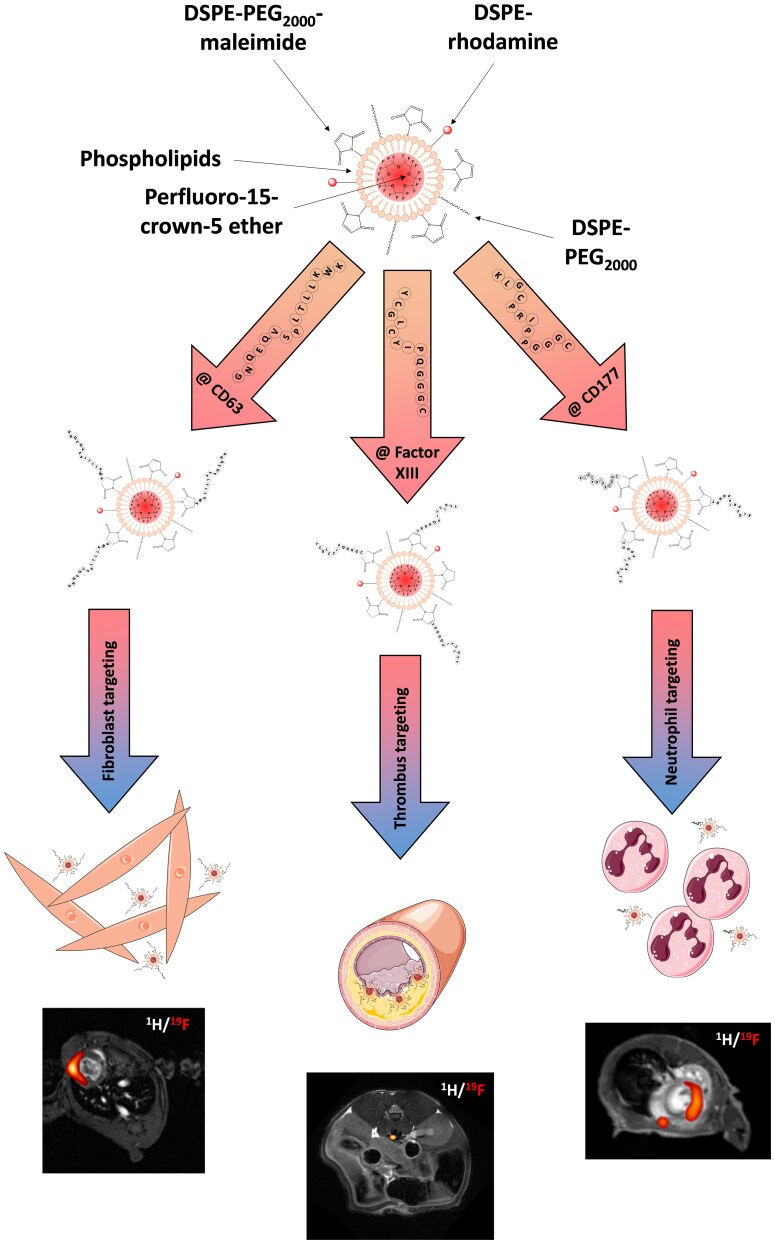
For active targeting approaches, FNPs with a functional maleimide group on their surface as well as a PEG chain are prepared. After coupling of different peptides (@ CD63, @ factor XIII, @ CD177) to the maleimide group on the surface of the FNPs they can be injected intravenously. With this approach it is possible to target activated cardiac fibroblasts (@ CD63), thrombi (@ factor XIII) or neutrophils (@ CD177). Adapted from Martinez *et al*. 2025 (left) [20], Temme *et al*. (middle) [21], and Bouvain *et al*. (right) [22].

Active targeting of FNPs makes use of the binding properties of specific ligands such as antibodies, peptides, sugars or nanobodies. These can be coupled to the FNP surface *via* functional groups. A common method is to couple the ligands to maleimide-functionalized lipids (Fig. 3, top), which is either incorporated directly into the FNPs or can be integrated into the pre-formulated FNPs at a later stage *via* the sterol-based post insertion technique (SPIT) [23]. In this way, a wide variety of cells can be targeted, provided, of course, that the ligand has a high specificity for the selected epitopes. In addition, it must first be ensured that those targeted FNPs are not also passively taken up by phagocytic cells. The common gold standard for protection of NPs against phagocytosis is surface PEGylation (Fig. 3, top). The long PEG chains prevent the binding of serum proteins, a process which normally leads to formation of a protein corona facilitating their phagocytosis by monocytes/macrophages. If serum proteins bind to the nanoemulsions through van der Waals or other electrostatic forces, the PEG chains are compressed, absorb energy, and then release it again by repelling the serum proteins [24]. Thus, PEGylation makes recognition of NPs by phagocytic cells more difficult [25]. Furthermore, the surface charge becomes more neutral, another factor that reduces non-specific uptake [26]. However, a potential drawback is, that repeated administration of PEGylated NPs can induce the formation of anti-PEG antibodies, which may lead to rapid particle clearance or, in severe cases, anaphylactic reactions [27]. Consequently, ongoing research is exploring alternative strategies, such as the use of CD47 as a ‘don’t eat me’ signal [28]. Another interesting approach is the utilization of zwitterionic coating of FNPs to prevent the building of a protein corona resulting in less uptake by the reticuloendothelial system (RES) [29, 30]. Beyond accumulation in the RES, it is important to note that most NPs, aside from their surface modifications, tend to accumulate in the spleen and liver, which poses a significant challenge for various diagnostic and therapeutic applications. It is therefore essential to reduce this enrichment as much as possible in order to also enable imaging/therapy in these organs without side effects in the future.

Despite these limitations, this approach enables the targeted labeling of specific cell populations or tissue structures. As an example, we demonstrated that the coupling of targeting peptides—such as those directed against α2-antiplasmin or fibrin—allows for the active targeting of acute and chronic thrombi. These can be detected *in vivo* using combined ^1^H/^19^F MRI [21] (Fig. 3, middle). α2-antiplasmin-decorated FNPs bind to fibrin through crosslinking by activated factor XIII during the early stages of thrombus formation and were administered to mice in which thrombi had been induced in the *vena cava* using FeCl_3_ (Fig. 3, bottom middle) [21]. Beyond this, within the cardiovascular field, several other applications were reported using targeted FNPs. Waters *et al*. [31] prepared α_v_β_3_-specific FNPs to image aortic valve disease in a rabbit model while Southworth *et al*. [32] designed VCAM-1-specific FNPs to target general inflammation in the context of atherosclerosis. Another important field of FNP-based molecular imaging is oncology. Several years ago, Bae *et al*. [33] employed folate receptor-targeted FNPs to visualize nasopharyngeal tumors. In addition, angiogenesis during tumor progression was successfully monitored in a brain tumor model using FNPs also functionalized with an α_v_β_3_-specific ligand [34].

In addition to this tissue selective approaches, distinct cell populations can also be visualized using this approach. We recently demonstrated that activated cardiac fibroblasts can be specifically labeled and subsequently detected within the infarcted heart (Fig. 3, bottom left) *via* upregulation of CD63 on their cell surface [20]. Moreover, by employing a peptide binding to CD177, we successfully labeled neutrophils in the bone marrow using targeted FNPs. Following the induction of acute experimental myocardial infarction, we were able to monitor the mobilization of neutrophils from the bone marrow and track their migration into the inflamed heart tissue *in vivo* [22] (Fig. 3, bottom right).

## Theranostics

Beyond ‘just’ imaging, the combination of imaging and direct therapeutic intervention—commonly referred to as theranostics—is an important development. This approach offers the advantage of not only visualizing biological processes but also inducing targeted functional changes within the affected tissue at the same time.

### Targeted theranostics and magnetic resonance imaging in general

MRI-based theranostics has been mostly used for preclinical cancer models but also for imaging and treatment of cardiovascular diseases. Similarly as pointed out above, the most widely used MRI CAs that were used for theranostics are IO-NPs, Mn or Gd, whereby Mn or Gd are either complexed in chelates or are also doped/encapsulated in NPs. In the following, we briefly describe some general aspects for theranostic applications with these CAs and then describe few examples of targeted theranostics in more detail. A broader overview about studies on MRI-based theranostics is provided in Table 1.

**Table 1.** Overview about theranostics in combination with ^1^H-based MRI contrast agents.

MR contrast	Target	Ligand	Therapeutic drug/therapy	Disease	Brief summary	Reference
IO-NPs	GLUT	Glucuronic acid	Iron oxide (hyperthermia)	Cancer	IO-NPs were functionalized with glucuronic acid (NPs@GlcA) to target glucose transporters (GLUT) expressed on brain endothelial cells and glioblastoma. Systemic administration of NPs@GlcA resulted in transcytosis *via* the blood brain barrier and accumulation in C6 glioblastoma in mice. Magnetic hyperthermia led to substantial decrease of tumor growth.	Caro *et al*. [35]
IO-NPs	Tumor	Cancer cell mem­brane (homotypic targeting)	Lycorine hydrochloride	Cancer	IO-NPs enwrapped by HT29 cell membrane and loaded with lycorine hydrochloride reduced growth of HT29 colorectal cancer in mice.	Li *et al*. [36]
IO-NPs	P-Selectin	Fucoidan	Fucoidan	Cancer	Fucoidan coated IO-NPs target P-selectin on the lung cancer cell line A549 leading to higher ROS-production, increased cytotoxicity and less migration/invasion *in vitro*.	Ho *et al*. [37]
IO-NPs	PDGFR	PDGF	Glucose oxidase/FePt	Cancer	Nanoliposomes targeted against platelet-derived growth factor receptor (PDGFR) that contain IO-NPs, glucose oxidase and platinum increased ROS production and induced apoptosis/ferroptosis. Treatment of mice with a breast cancer xenograft (MDA-MB-231) reduced tumor size.	Zhang *et al*. [38]
IO-NPs	Sialic acid	Phenylboronic acid	Phototherapy	Cancer	Targeting of sialic acid on tumor cells with IO-NP-containing particles that led to enhanced photothermal response and strongly reduced tumor volume (ovarian cancer) in mice.	Lu *et al*. [39]
IO-NPs	α_v_β_3_-Integrin	RGD peptide	Doxorubicin/indocyanine green	Cancer	IO-NPs functionalized with RGD to target α_v_β_3_-integrin on U87MG tumor cells and doxorubicin/indocyanine green for enhanced photothermal and chemotherapy that strongly reduced tumor size in mice.	He *et al*. [40]
IO-NPs	Transferrin receptor	Transferrin	Doxorubicin/high intensity ultrasound	Cancer	Iron-platinum nanoparticles are encapsulated together with doxorubicin in nanobubbles (NB) functionalized with transferrin. High intensity focused ultrasound breaks the NBs, opens the blood brain barrier and enables the targeting of the glioblastoma in mice.	Chan *et al*. [41]
IO-NPs	Fibronectin	CREKA peptide	SQ photosensitizer near infrared = NIR	Cancer	IO-NPs functionalized with the peptide CREKA to target fibronectin in the ECM of tumors as well as the photosensitizer SQ. Growth of MDA-MB-231 tumors was clearly reduced in mice treated with these NPs in combination with NIR laser irradiation.	Wang *et al*. [42]
IO-NPs	Hsp70	GranzymB	GrB/Hsp70 induction of apoptosis	Cancer	Hsp70 containing IO-NPs that bind to GranzymB on the surface of tumor cells which is followed by internalization and induction of apoptosis. Treatment of mice with HT1339 lung tumors or U87 glioma with Hsp70-IO-NPs increased the overall survival time.	Shevtzov *et al*. [43]
IO-NPs	Fibrin/ Fibronectin	CREKA peptide	Iron oxide (hyperthermia)	Cancer	Small IO-NPs with CREKA peptide as targeting moiety in combination with magnetic hyperthermia resulted in strongly reduced tumor growth in a murine 4T1 breast tumor model.	Du *et al*. [44]
IO-NPs	CD44	Hyaluronic acid	Chlorin e6 photodynamic therapy	Cancer	IO-NPs with hyaluronic acid on the surface to target CD44 were further functionalized with the photosensitizer chlorin e6 which resulted in high toxicity after laser irradiation in BL6F1 cell *in vitro*.	Dehvari *et al*. [45]
IO-NPs	Folic acid receptor	Folic acid	Doxorubicine released in redox response	Cancer	Micellar nanoparticles with folic acid as targeting ligand and IO-NPs and doxorubicin. Intravenous injection into HeLa tumor bearing mice massively reduced tumor size particularly in combination with magnetic targeting.	Wei *et al*. [46]
IO-NPs	EGFR	Anti-EGFR mAb	Magnetic resonance-guided focused ultrasound surgery (MRgFUS)	Cancer	Anti-EGF receptor antibody (Cetuximab) was attached to IO-NPs and used to treat nude rats with H460 lung cancer xenografts with MRgFUS. Application of anti-EGFR-IO-NPs reduced energy for MRgFUS and led to smaller tumor sizes.	Wang *et al*. [47]
IO-NPs	Urokinase plasminogen activator re­ceptor (uPAR)	Amino-terminal fragment of urokinase plasminogen activator (ATF)	Cisplatin	Cancer	Milk protein coated IO-NPs loaded with Cisplatin and functionalized with ATF to bind to uPAR. Treatment of mice with orthotropic pancreatic tumors in mice with these IO-NPs strongly reduced the tumor weight.	Huang *et al*. [48]
Mn	α_v_β_3_-Integrin	cRGDyK peptide	Mn^2+^−STING activation and anti-PD-L1	Cancer	MnFe_2_O_4_ NPs labeled with RGD bind to α_v_β_3_-integrins strongly expressed in many tumors. Treatment of mice with RGD-MnFe_2_O_4_ NPs inducted the STING pathway and resulted in a reduced tumor growth (B16F10, CT26, 4T1) in particularly in combination with αPD-L1 therapy.	Shi *et al*. [49]
Mn	Tumor tissue	Cancer cell mem­brane (homotypic targeting)	Doxorubicin	Cancer	Manganese dioxide coated gold nanorods loaded with doxorubicin were enwrapped in cancer cell membrane. Application in 4T1 breast tumor-bearing mice enhanced the efficacy of photothermal therapy and led to reduced tumor size.	Zhang *et al*. [50]
Mn	Transferrin receptor	Apoferritin	Doxorubicin	Cancer	MnO_2_ apoferritin NPs that target the transferrin receptor were loaded with doxorubicin. Treatment of 4T1 tumor bearing mice led to a strongly reduced tumor growth which was supposed to be based on a synergistic effect of MnO_2_ to increase the oxygen level and doxorubicin.	Veroniaina *et al*.[51]
Gd	Folate receptor	Folic acid	Titanium (Ti)/ROS-production	Cancer	NPs that incorporated Gd, Ti, and a near infrared dye into tannic acid (TA). NPs were further functionalized with folic acid to target the FA-receptor. Interestingly, administration of Gd-Ti-FA-TA NPs to mice with 4T1 tumors reduced tumor progression only in combination with ^18^FDG.	Huang *et al*. [52]
Gd	CD44	Hyaluronic acid (HA)	Rosuvastatin (RST)	Atherosclerosis	Cerasomes modified with HA and RST selectively target CD44-positive cells in atherosclerotic plaques. Injection of these NPs in ApoE^−/−^ mice on a high fat diet reduced plaque burden *via* immunmodulatory effects.	Ma *et al*. [53]
Gd	VEGF receptor	VEGF mimetic peptide	QK peptide	Myocardial infarction	Gd-doped carbon dots (CD) were functionalized with the VEGF mimetic peptide QK that stimulates vascularization. Intravenous application of Gd-CD-QK NPs reduced adverse cardiac remodeling after myocardial ischemia/reperfusion injury in mice.	Li *et al*. [54]
Gd	α_v_β_3_-Integrin	RGD peptide	Chlorin e6 (Ce6)	Cancer	DSPE-PEG_2000_ was modified with a peptide and DTPA (diethylenetriamine pentaacetic acid = Gd chelator) to generate organic NPs that contain Ce6 and Gd. These DSPM@Ce6@Gd NPs in combination with photodynamic therapy strongly inhibited the growth of A549 tumors in mice.	Yang *et al*. [55]
Gd	PS receptor	Phosphatidylserine (PS)	Liraglutide (GLP-1R agonist)	Atherosclerosis	Multilayered NPs decorated with PS and coated with PEG and gelatin. Liraglutide was inserted between the core and the gelatin layer. Intravenous injection of NPs into ApoE^−/−^ mice treated with a western diet reduced lesion size and the macrophage content.	Maiseyeu *et al*. [56]
Gd	α_v_β_3_-Integrin	RGD peptide	Camptothecin (CPT)/NIR-photosensitizer (PPa-SH)	Cancer	A theranostic nanoprobe (Co-NP-RGD) with RGD-peptides for tumor targeting, a near infrared photosensitizer and CPT. Co-NP-RGD disassemble in the tumor releasing CPT/PPa-SH for chemo- and photodynamic therapy. Repetitive treatment of U87MG glioma mice with Co-NP-RGD and irradiation reduced tumor growth and increased the survival of the animals.	An *et al*. [57]
Gd	Methotrexat (MTX)	Folate receptor	Artesunate (ASA)/Methotrexat	Cancer	MTX, ASA and Gd were assembled into NPs (ASA-MTX-Gd^III^) for combined chemo-dynamics (ROS-production) and chemotherapy. NPs are taken up *via* the folate receptor and release MTX/ASA within acidic endosomes. Treatment of nude mice with HeLa tumors nearly completely inhibited tumor progression.	Fan *et al*. [58]
Gd/IO	α_v_β_3_-Integrin	RGD peptide	Docetaxel (DTX)	Cancer	Preparation of a Gd-containing IO-nanocluster further functionalized with RGD for targeting the integrin α_v_β_3_ and docetaxel for therapy (cRGD-GdIO-DTX). NPs degrade in the acidic tumor environment and release DTX. Systemic application of cRGD-GdIO-DTX to mice with implanted Panc-1 pancreatic cancer cells strongly impaired tumor growth.	Wang *et al*. [59]

#### Iron oxide nanoparticles

Analogous to FNPs, the surface of IO-NPs can also be functionalized with targeting ligands like antibodies, antibody-derivatives, peptides or other small molecular ligands and have similarly been used for targeted cell tracking or thrombus imaging [60] as well as been doped or functionalized with drugs for therapeutic purposes [61]. For the latter, applications for treatment of anemia, chemotherapy, hyperthermia, chemodynamic-, or immunotherapy have been reported [11]. For example, ferumoxytol is approved for treatment of patients with anemia and chronic kidney diseases [62]. Another interesting observation is that exposure of tumor-associated macrophages to IO-NPs shifts their polarization towards an pro-inflammatory phenotype with elevated production of reactive oxygen species (ROS) that supports the immune response against cancer [63]. Other therapeutic approaches are magnetic hyperthermia where an alternating external magnetic field produces heat that enhances the local immune response against tumors and also drives tumor-cells into apoptosis [64].

Beyond those rather non-specific approaches, targeting of IO-NPs enables a more directed delivery of the imaging agent as well as the therapeutic substance to a certain site of the body or specific cell-types or structures. This enhances the local concentration of the drug, increases the efficiency and also reduces side effects. Active targeting of IO-NPs can be conducted by functionalization of the nanoparticle surface with targeting ligands or magnetic targeting. For magnetic drug targeting a strong magnet is placed above the target site that attracts and locally fixes the IO-NPs and thereby enhances the local accumulation of the drug-loaded IO-NPs [64]. On the other hand, for specific targeting, Schleich *et al.* [65] modified IO-containing nanoparticles with paclitaxel (PTX) and a tripeptide composed of arginine-glycine-aspartic acid (RGD) for targeting α_v_β_3_-integrins. Paclitaxel is a chemotherapeutic drug that inhibits the proliferation of tumor cells by binding to β-tubulin which blocks the degradation of microtubuli and arrests the cells in the G2-/M-phase. The linear RGD peptide motive is found in many molecules involved in cell adhesion or signaling like fibronectin, vitronectin, or fibrinogen and binds to different integrin molecules [66]. Due to the fact that integrins like α_v_β_3_ or α_v_β_5_ are overexpressed in the vasculature of tumors, but are also found on the surface of tumor cells, RGD-targeting has been widely used for tumor imaging, but also for targeted drug delivery. Here, CT26-tumor bearing mice were treated with RGD-targeted SPIO/PTX-NP (or control NPs) which revealed that RGD-targeting in combination with magnetic targeting strongly reduced the tumor growth and elevated the survival rate. Interestingly, RGD- or magnetic-targeting had a similar effect that was higher as untargeted controls. This was in agreement with T2-weighted (T2w) MRI of the tumor which showed dark spots with heterogeneous distribution within the tumor after application of RGD-targeted NPs, magnetic targeting or both.

In a more recent study, Wang *et al*. [59] prepared IO-NPs that contain docetaxel as anti-cancer drug and a cyclic version of the RGD peptide (cRGD) for integrin targeting on pancreatic cancer cells. Of note, the authors also incorporated Gd to generate a T1-T2 dual-mode imaging (Gd/IO) nanocluster. Docotaxel (DTX) is also an anti-cancer drug that belongs to the same group of molecules as paclitaxel (taxanes). It has a similar mode of action and blocks the proliferation of cells by binding and stabilizing microtubuli and by induction of apoptosis. *In vitro*, application of cRGD-GdIO-DTX targeted NPs had a stronger negative effect on cell viability of Panc-1 cells than untargeted GdIO-DTX. Quite impressive results were obtained *in vivo*, where application cRGD-GdIO-DTX nearly completely inhibited the growth of subcutaneously implanted Pac-1 tumor cells in nude mice. Finally, MRI revealed that injection of cRGD-GdIO-DTX resulted in enhanced T1w and darkened T2w images.

IO-NPs have also been utilized in regenerative medicine for example to load and track therapeutic cells. De Vries *et al.* [67] labeled autologous immature dendritic cells with SPIOs and reinjected them into the lymph nodes of stage-III melanoma patients and subsequently investigated the localization and migration by scintigraphy or MRI. Interestingly, MRI was superior to assess the precise localization of DCs in or outside of lymph nodes which is important for the proper induction of an immune response. Furthermore, iron oxide labeled scaffolds (e.g. collagen or vascular grafts) have been developed where the proper localization as well was the stability of the device can be analyzed over time by MRI [68, 69].

#### Manganese

Mn-based MRI has been used for diagnostic purposes (see above) but also for therapeutics and theranostics [70]. Mn^2+^ in the cytosol can for example induce Fenton-like reactions that generate ROS and therefore has been used for chemodynamic therapy [71]. Mn has also been shown to stimulate the innate immune system by activation of the cGAS-STING pathway [72] that can be exploited for immunotherapy [70]. Furthermore, Mn-NPs can also be loaded with drugs for therapy. For example, Pan *et al.* generated BSA (bovine serum albumine)-MnO_2_ NPs functionalized with paclitaxel for chemotherapy against cancer [73].

A targeted approach has been conducted by Zhou *et al.* [74] who generated Mn-CaPO_4_ NPs loaded with doxorubicin that were functionalized with the EGFR-specific antibody C225. These NPs were efficiently internalized by EGFR-positive BxPC-3 cancer cells and exhibited toxic effects after uptake. Conjugation of mAb C225 to the nanoparticles further enhanced the cellular toxicity about 1.5-fold.

Shi *et al*. 2024 generated RGD-targeted magnetic Mn-containing ferrite NPs (MnFe_2_O_4_) and exploited their utility for anti-cancer therapy. The authors found that RGD-MnFe_2_O_4_ showed increased accumulation in tumors and was retained inside the tumor tissue for longer periods of time. Moreover, these nanoparticles stimulated the cGAS-STING pathway in an immortalized dendritic cell line which was assessed by analysis of phosphorylated TBK1 and the translocation of STING from the cytoplasm to the nucleus. Treatment of several tumors revealed that exposure to RGD-NPs or anti-PDL1 decreased tumor growth, which was strongly increased when RGD-NPs were used in combination with anti-PDL1. Furthermore, treatment of mice bearing CT26 tumors with RGD-NPs plus anti-PDL1 resulted in higher numbers of CD8^+^/CD4^+^ T cells, activated CD69^+^ T cells and CD86^+^ dendritic cells in the tumor tissue.

#### Gadolinium

In addition to the normal use of GBCAs (e.g. first pass or late Gd enhancement (LGE)) they have also been combined with therapeutic molecules for MR theranostics [75]. Wang *et al*. [59] generated iron- and Gd-containing nanocluster that were functionalized with the peptide cRGD as well as the anti-tumor drug docetaxel (DTX → cRGD-GdIO-DTX) for targeting of α_v_β_3_ integrins on the surface of Panc-1 pancreatic cancer cells. Here, the inhibition of proliferation of Panc-1 or SW1990 cells was much stronger than that by untargeted particles. Furthermore, in nude mice with a human pancreatic cancer xenograft, cRGD-GdIO-DTX slowed tumor development and in parallel showed signal enhancement in MRI. In another study, Ma *et al*. [53] generated hyaluronic acid (HA) containing cerasomes (HA-CC) that selectively recognize CD44-positive cells within atherosclerotic plaques. Cerasomes are liposome-like NPs that were generated by a combination of organoalkoxysilane lipid 1, phosphoethanolamine, and phosphatidylcholine. HA was linked to the surface of the nanoarticles and for therapeutic purposes, the authors also incorporated the rosuvastatin (RST) into the hydrophobic layer to produce HA-CC-RST. RST is a hydrophobic drug that belongs to the group of statins and inhibits the biosynthesis of cholesterol by blocking the conversion of HMG-CoA to mevalonate *via* the enzyme HMG-CoA reductase. Importantly, recent data indicates that statins also have anti-inflammatory or immunomodulatory effects that could reduce the plaque burden [76]. To visualize the accumulation of these nanoparticles within atherosclerotic plaques, Gadodiamide was also inserted into the CCs. T1w MRI revealed an increased accumulation of HA-CCs in the aortic wall of ApoE^−/−^ mice fed with a high-fat diet leading to an enhanced MRI contrast in atherosclerotic plaques compared to non-targeted CCs. Furthermore, treatment of ApoE^−/−^ mice with HA-CC-RST significantly decreased the amount of atherosclerotic lesions by ∼56% and also the overall proinflammatory profile. Of note, HA-CC-RST was more effective than the systemic administration of RST.

Taken together, a wide range of theranostic applications has already been investigated using conventional MRI CAs, but as mentioned above, none of the three classic agents (IO-NP, GBCAs, manganese) are inert in themselves, which means that they can trigger (undesirable) signal cascades.

### FNP-based theranostics

In addition to the broad potential of theranostic applications, numerous approaches have recently emerged that utilize ¹⁹F MRI for diagnostic purposes. One of the main challenges in this context lies in the physicochemical nature of FNPs. Due to their fluorophilic and hydrophobic core, water-insoluble drugs cannot simply be incorporated *via* conventional emulsification techniques. However, this limitation has been successfully addressed through the development of triphasic systems [77–79] or double emulsions [80–82], which allow for the integration of otherwise incompatible compounds. This advancement has already enabled the successful implementation of several theranostic strategies. For instance, small molecules such as rapamycin have been employed to treat muscular dystrophy [83], while celecoxib has been used to target neuroinflammation [84]. In addition to small molecules, other bioactive compounds have also been incorporated into FNPs. For example, siRNA targeting the STAT3 signaling pathway has shown therapeutic efficacy in both idiopathic pulmonary fibrosis [85] and lung metastatic osteosarcoma [86]. Furthermore, proteins and peptides, such as those targeting lactate oxidase, have been utilized in the treatment of breast cancer [87]. Recently, Vu-Quang *et al*. made use of dual-labeled near infrared and ^19^F NPs for delivery of doxorubicin to nasopharyngeal tumors *via* folate receptor targeting. They could show that killing of cancer cells was significantly improved in comparison to non-folate targeted nanoparticles [88].

A completely different strategy has been explored through the combination of FNPs with high-intensity focused ultrasound (HIFU), enabling both imaging and therapeutic applications. This is used to treat tumors and has already been approved by the FDA for the treatment of certain types of cancer, such as prostate cancer [89]. The method is based on the bundling of sound waves to destroy tissue through overheating. Current research is focusing on microbubbles to reduce treatment time and the acoustic energy. However, microbubbles have only a blood half-life of several minutes which significantly complicates the infusion into the tumor. For this reason, Shin *et al*. used FNPs. This enables precise imaging of the tumor through accumulation of the FNPs and also allows the concentration to be determined on site. It was found that a concentration of 2–3 mg/ml FNPs had the most promising effect in combating the tumor [90]. An overview of all studies is given in Table 2.

**Table 2.** Overview of theranostic ^19^F-based approaches.

MR-Contrast	Target	Ligand	Therapeutic drug/therapy	Disease	Brief summary	Reference
PFOB			Rapamycin	Duchenne muscu­lar dystrophy	Rapamycin-loaded NPs restore autophagy and improve muscle and cardiac function in mdx mice beyond conventional treatment.	Bibee *et al*. [83]
PFOB			Celecoxib	Neuroinflammation	Celecoxib nanoemulsions target macrophages, reduce PGE2 and inflammation, and relieve neuropathic pain with minimal systemic exposure.	Janjic *et al*. [84]
PFOB			siRNA	Pulmonary fibrosis	PFOB-NE enhance pulmonary siRNA delivery, effectively silencing STAT3/CXCR4 and inhibiting lung fibrosis progression.	Ding *et al*. [85]
HFBA			siRNA	Osteosarcoma	F-PCX@PFC nanoemulsions deliver siSTAT3 to lungs, inhibit immunosup­pression, and prolong survival in metastatic osteosarcoma models.	Li *et al*. [86]
PFTBA	PD-L1	PD-1	Membrane vesicles of salmonella typhimurium	Tumor	Hybrid membrane-coated nanoemulsions enhance targeted PD-L1 blockade, photothermal therapy, and T cell immunity to inhibit tumor growth and metastasis.	Zhang *et al*. [87]
PFOB	Folate receptor	Folic acid	Doxorubicin	Nasopharyngeal tumor	Folate-targeted PLGA-PEG NPs co-deliver Dox and imaging agents, enabling targeted chemotherapy and dual ^19^F MRI/NIR tumor tracking.	Vu-Quang *et al*. [88]
F68			High intensity focused ultrasound	Tumor	^19^F MRI tracks PFC-NE in tumors, optimizing HIFU ablation efficacy and necrosis based on PFC-NE accumulation.	Shin *et al*. [90]
PFOB	Integrin	α_V_β_3_	Rapamycin	Stenosis	α_v_β_3_-targeted rapamycin NPs reduce vascular stenosis after balloon injury without impairing endothelial healing in rabbits.	Cyrus *et al*. [91]

#### Simultaneous imaging and functional modification of neutrophils

**Figure 4.**
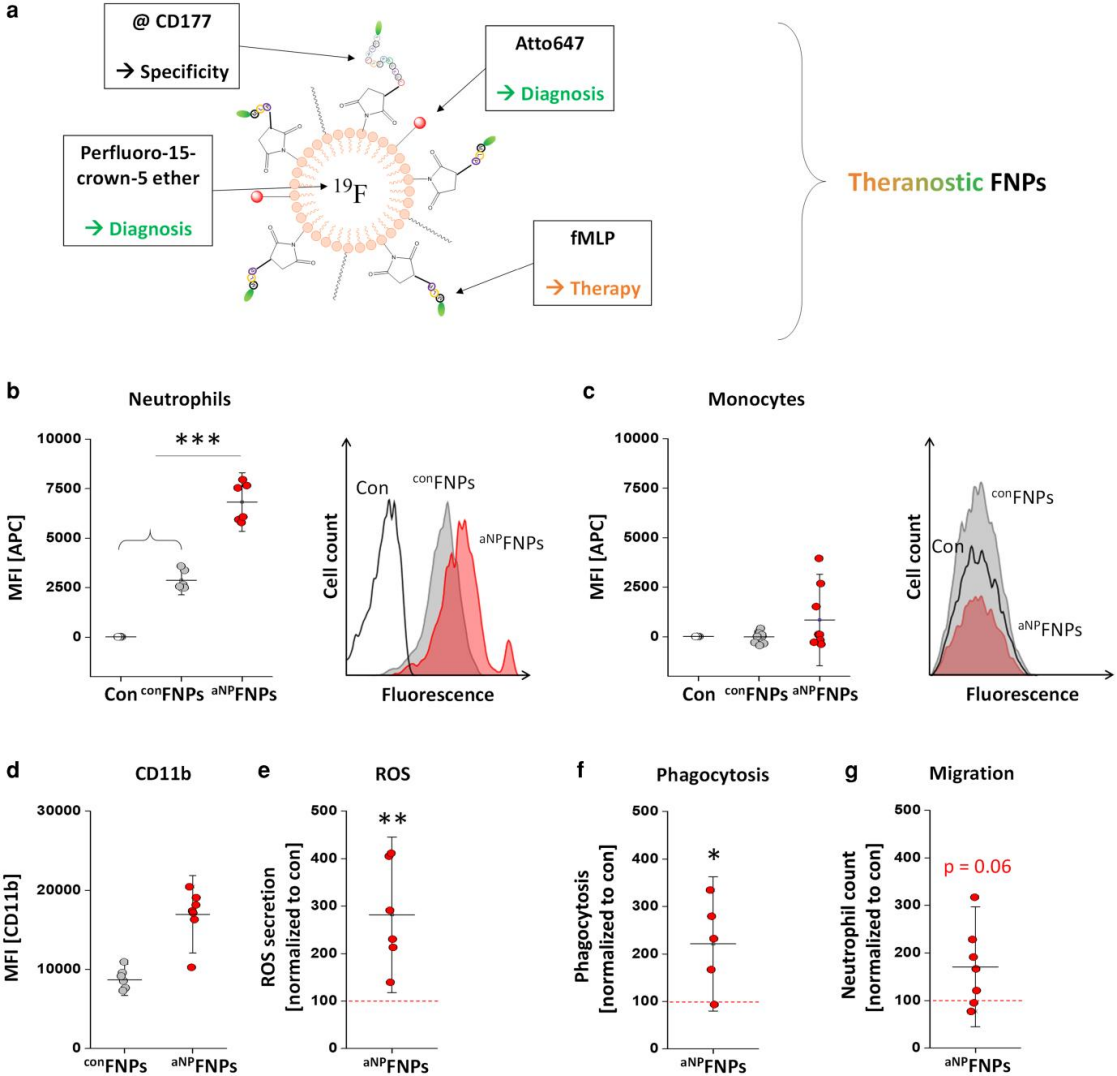
a**)** For combining therapy and diagnosis we modified the neutrophil-specific FNPs with an additional fMLP for functional activation of neutrophils to form ^aNP^FNPs. b + c**)** Although fMLP binds to the FPR1 receptor, which is expressed not exclusively by neutrophils but also monocytes, the ^aNP^FNPs still predominantly target neutrophils. Beside their binding we could detect an increase in the surface activation marker CD11b (d) due to the treatment as well as increased production of reactive oxygen species (e), phagocytosis of *E. coli* particles (f) and stronger migratory capacities (g). Adapted from Bouvain *et al.* [92].

Most recently, we have further developed the neutrophil imaging technology into a theranostic approach. In order to advance the pure targeting of neutrophils into a theranostic approach, we additionally equipped the surface of the FNPs with fMLP to generate neutrophil-activating ^aNP^FNPs. The fMLP moiety activates cells *via* the G protein-coupled receptor FPR1. Although FPR1 is not exclusively expressed on neutrophils, our data show that CD177-mediated binding predominates, preserving high targeting specificity. Importantly, we investigated whether this system could also induce neutrophil activation. Indeed, incubation with ^aNP^FNPs increased the expression of activation markers, enhanced reactive oxygen species production, and promoted both *E. coli* phagocytosis and migration into inflamed tissue (Fig. 4) [92].

**Figure 5.**
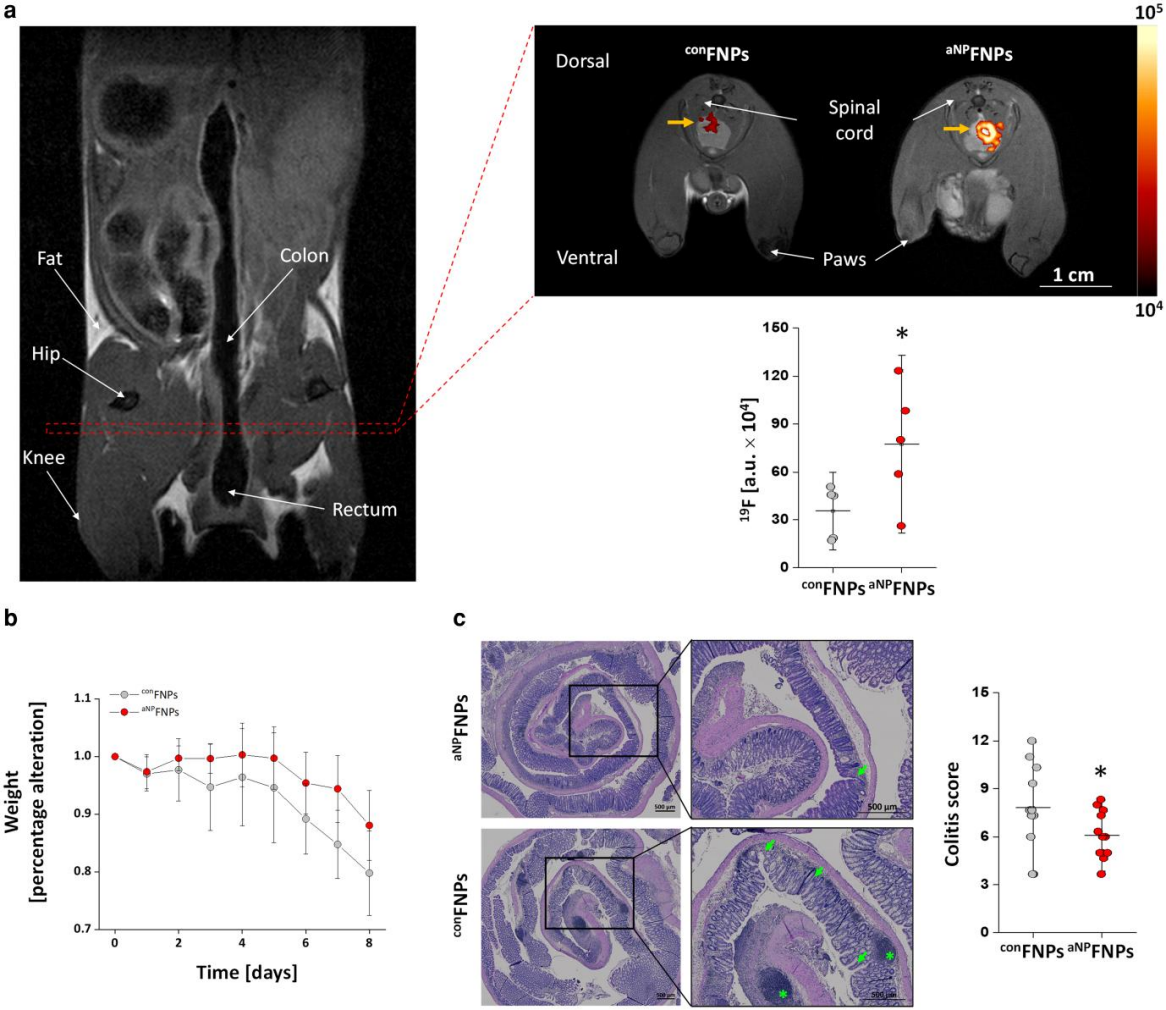
a) Shown is the intestinal tract of mice suffering from DSS-induced acute colitis. Neutrophils in the inflamed intestine can be detected by injecting neutrophil-specific FNPs (^con^FNPs) and the theranostic variant, which was additionally functionalized with fMLP on its surface (^aNP^FNPs). Treatment with ^aNP^FNPs not only shows increased migration of neutrophils to the affected area, but also an improved outcome after colitis. This was quantified on one hand by lower weight loss over time (b) and on the other hand by a significantly lower colitis score (c), which reflects the severity of colitis based on histological evaluation. Adapted from Bouvain *et al.* [92].

After confirming that this approach selectively activates neutrophils while leaving other immune cells largely unaffected, we evaluated its therapeutic potential in an established disease model. In acute colitis—characterized by intestinal epithelial cell death and subsequent bacterial translocation—neutrophils are among the first responders. *Via* non-invasive mapping we could find strong neutrophil influx into the affected gut. Pre-activation with ^aNP^FNPs markedly improved disease outcome, as evidenced by reduced weight loss and significantly improved histological colitis scores compared to control-treated mice (Fig. 5) [92].

## Future perspective

^19^F MRI for molecular imaging purposes has developed at remarkable speed. Since the first demonstrations of *in vivo* imaging of inflammatory processes nearly two decades ago, the field has advanced enormously. Today, it is possible not only to visualize distinct immune cell populations *in vivo* but also to simultaneously address multiple targets by employing different FNP formulations. Moreover, the field has evolved beyond pure diagnostics: fluorine-based nanoplatforms can now be harnessed for therapeutic purposes, enabling functional modulation of immune cells and targeted intervention in disease processes. With continuous advances, the implementation of this technology in clinical practice is getting closer and promises a new era of precise and personalized theranostics.

## Data Availability

All data supporting the findings of this study are available within the article.

## References

[1] Weiss JN, Goldhaber JI, Ji S. Oxygen free radicals in the pathophysiology of myocardial ischemia/reperfusion. In: Tarr M, Samson F (eds.), Oxygen Free Radicals in Tissue Damage. Boston, MA: Birkhäuser, 1993, 250–66.

[2] Aronova A, Tosato F, Naser N et al Innate immune pathways in atherosclerosis—from signaling to long-term epigenetic reprogramming. Cells 2023;12:2359. 10.3390/cells12192359 37830572 PMC10571887

[3] Khandaker GM, Cousins L, Deakin J et al Inflammation and immunity in schizophrenia: implications for pathophysiology and treatment. Lancet Psychiatry 2015;2:258–70. 10.1016/S2215-0366(14)00122-9 26359903 PMC4595998

[4] Shoenfeld Y, Gershwin ME. Contemporary Challenges in Autoimmunity, Vol. 1173. Boston, MA: John Wiley & Sons, 2009.

[5] Bianchi ME . DAMPs, PAMPs and alarmins: all we need to know about danger. J Leukoc Biol 2007;81:1–5. 10.1189/jlb.0306164 17032697

[6] Quattrocchi CC, Rovira À, van der Molen AJ et al ESR essentials: gadolinium-wise MRI—practice recommendations by the European Society for Magnetic Resonance in Medicine and Biology. Eur Radiol 2025;35:3347–53. 10.1007/s00330-024-11214-4 39702634 PMC12081572

[7] Hasebroock KM, Serkova NJ. Toxicity of MRI and CT contrast agents. Expert Opin Drug Metab Toxicol 2009;5:403–16. 10.1517/17425250902873796 19368492

[8] Kim H-K, Lee GH, Chang Y. Gadolinium as an MRI contrast agent. Future Med Chem 2018;10:639–61. 10.4155/fmc-2017-0215 29412006

[9] Niu D, Luo X, Li Y et al Manganese-loaded dual-mesoporous silica spheres for efficient T1- and T2-weighted dual mode magnetic resonance imaging. ACS Appl Mater Interfaces 2013;5:9942–8. 10.1021/am401856w 24059807

[10] Ding Z, Liu X, Jiang H et al A refined TTC assay precisely detects cardiac injury and cellular viability in the infarcted mouse heart. Sci Rep 2024;14:25214. 10.1038/s41598-024-76414-w 39448689 PMC11502796

[11] Dadfar SM, Roemhild K, Drude NI et al Iron oxide nanoparticles: diagnostic, therapeutic and theranostic applications. Adv Drug Deliv Rev 2019;138:302–25. 10.1016/j.addr.2019.01.005 30639256 PMC7115878

[12] Neuwelt A, Sidhu N, Hu C-AA et al Iron-based superparamagnetic nanoparticle contrast agents for MRI of infection and inflammation. AJR Am J Roentgenol 2015;204:W302–13. 10.2214/AJR.14.12733 25714316 PMC4395032

[13] Masthoff M, Freppon FN, Zondler L et al Resolving immune cells with patrolling behaviour by magnetic resonance time-lapse single cell tracking. eBioMedicine 2021;73:103670. 10.1016/j.ebiom.2021.103670 34742131 PMC8581510

[14] Flögel U, Ding Z, Hardung H et al In vivo monitoring of inflammation after cardiac and cerebral ischemia by fluorine magnetic resonance imaging. Circulation 2008;118:140–8. 10.1161/CIRCULATIONAHA.107.737890 18574049 PMC2735653

[15] Ebner B, Behm P, Jacoby C et al Early assessment of pulmonary inflammation by 19F MRI in vivo. Circ Cardiovasc Imaging 2010;3:202–10. 10.1161/CIRCIMAGING.109.902312 20061515 PMC3138443

[16] Ahrens ET, Flores R, Xu H et al *In vivo* imaging platform for tracking immunotherapeutic cells. Nat Biotechnol 2005;23:983–7. 10.1038/nbt1121 16041364

[17] Flögel U, Su S, Kreideweiß I et al Noninvasive detection of graft rejection by in vivo (19) F MRI in the early stage. Am J Transplant 2011;11:235–44. 10.1111/j.1600-6143.2010.03372.x 21214858

[18] Temme S, Jacoby C, Ding Z et al Technical advance: monitoring the trafficking of neutrophil granulocytes and monocytes during the course of tissue inflammation by noninvasive 19F MRI. J Leukoc Biol 2014;95:689–97. 10.1189/jlb.0113032 24319285

[19] Ding Z, Temme S, Quast C et al Epicardium-derived cells formed after myocardial injury display phagocytic activity permitting in vivo labeling and tracking. Stem Cells Transl Med 2016;5:639–50. 10.5966/sctm.2015-0159 27057005 PMC4835243

[20] Euan Martínez AA, Bergmann AK, Tellkamp F et al CD63 as novel target for nanoemulsion-based 19F MRI imaging and drug delivery to activated cardiac fibroblasts. Theranostics 2025;15:1–18. 10.7150/thno.96990 39744226 PMC11667234

[21] Temme S, Grapentin C, Quast C et al Noninvasive imaging of early venous thrombosis by 19F magnetic resonance imaging with targeted perfluorocarbon nanoemulsions. Circulation 2015;131:1405–14. 10.1161/CIRCULATIONAHA.114.010962 25700177

[22] Bouvain P, Ding Z, Kadir S et al Non-invasive mapping of systemic neutrophil dynamics upon cardiovascular injury. Nat Cardiovasc Res 2023;2:126–43. 10.1038/s44161-022-00210-w 39196054 PMC11357992

[23] Temme S, Baran P, Bouvain P et al Synthetic cargo internalization receptor system for nanoparticle tracking of individual cell populations by fluorine magnetic resonance imaging. ACS Nano 2018;12:11178–92. 10.1021/acsnano.8b05698 30372619

[24] Lee JH, Park JW, Lee HB. Cell adhesion and growth on polymer surfaces with hydroxyl groups prepared by water vapour plasma treatment. Biomaterials 1991;12:443–8. 10.1016/0142-9612(91)90140-6 1892978

[25] Hak S, Helgesen E, Hektoen HH et al The effect of nanoparticle polyethylene glycol surface density on ligand-directed tumor targeting studied in vivo by dual modality imaging. ACS Nano 2012;6:5648–58. 10.1021/nn301630n 22671719 PMC3389615

[26] He C, Hu Y, Yin L et al Effects of particle size and surface charge on cellular uptake and biodistribution of polymeric nanoparticles. Biomaterials 2010;31:3657–66. 10.1016/j.biomaterials.2010.01.065 20138662

[27] Kozma GT, Shimizu T, Ishida T et al Anti-PEG antibodies: properties, formation, testing and role in adverse immune reactions to PEGylated nano-biopharmaceuticals. Adv Drug Deliv Rev 2020;154–155:163–75. 10.1016/j.addr.2020.07.024 32745496

[28] Vandchali NR, Moadab F, Taghizadeh E et al CD47 functionalization of nanoparticles as a poly(ethylene glycol) alternative: a novel approach to improve drug delivery. Curr Drug Targets 2021;22:1750–9. 10.2174/1389450122666210204203514 33563192

[29] Choi HS, Gibbs SL, Lee JH et al Targeted zwitterionic near-infrared fluorophores for improved optical imaging. Nat Biotechnol 2013;31:148–53. 10.1038/nbt.2468 23292608 PMC3568187

[30] Wei H, Insin N, Lee J et al Compact zwitterion-coated iron oxide nanoparticles for biological applications. Nano Lett 2012;12:22–5. 10.1021/nl202721q 22185195 PMC3278278

[31] Waters EA, Chen J, Allen JS et al Detection and quantification of angiogenesis in experimental valve disease with integrin-targeted nanoparticles and 19-fluorine MRI/MRS. J Cardiovasc Magn Reson 2008;10:43. 10.1186/1532-429X-10-43 18817557 PMC2561020

[32] Southworth R, Kaneda M, Chen J et al Renal vascular inflammation induced by western diet in ApoE-null mice quantified by 19F NMR of VCAM-1 targeted nanobeacons. Nanomedicine 2009;5:359–67. 10.1016/j.nano.2008.12.002 19523428 PMC2780462

[33] Bae PK, Jung J, Lim SJ et al Bimodal perfluorocarbon nanoemulsions for nasopharyngeal carcinoma targeting. Mol Imaging Biol 2013;15:401–10. 10.1007/s11307-013-0622-2 23508465

[34] Giraudeau C, Geffroy F, Mériaux S et al 19F molecular MR imaging for detection of brain tumor angiogenesis: in vivo validation using targeted PFOB nanoparticles. Angiogenesis 2013;16:171–9. 10.1007/s10456-012-9310-0 23053783

[35] Caro C, Paez-Muñoz JM, Pernía Leal M et al Metabolically-driven active targeting of magnetic nanoparticles functionalized with glucuronic acid to glioblastoma: application to MRI-tracked magnetic hyperthermia therapy. Adv Healthc Mater 2025;14:2404391. 10.1002/adhm.202404391 39578332

[36] Li J, Lin C, Zhu Y et al Colorectal cancer cell membrane biomimetic ferroferric oxide nanomaterials for homologous bio-imaging and chemotherapy application. Med Oncol 2023;40:322. 10.1007/s12032-023-02175-7 37801170

[37] Ho T-L, Mutalik C, Rethi L et al Cancer-targeted fucoidan-iron oxide nanoparticles for synergistic chemotherapy/chemodynamic theranostics through amplification of P-selectin and oxidative stress. Int J Biol Macromol 2023;235:123821. 10.1016/j.ijbiomac.2023.123821 36870633

[38] Zhang C, Leng Z, Wang Y et al PDGFB targeting biodegradable FePt alloy assembly for MRI guided starvation-enhancing chemodynamic therapy of cancer. J Nanobiotechnology 2022;20:264. 10.1186/s12951-022-01482-x 35672821 PMC9172083

[39] Lu H, Xu J, Yang J et al On-demand targeting nanotheranostics with stimuli-responsive releasing property to improve delivery efficiency to cancer. Biomaterials 2022;290:121852. 10.1016/j.biomaterials.2022.121852 36270058

[40] He T, He J, Younis MR et al Dual-stimuli-responsive nanotheranostics for dual-targeting photothermal-enhanced chemotherapy of tumor. ACS Appl Mater Interfaces 2021;13:22204–12. 10.1021/acsami.1c03211 33956444

[41] Chan M-H, Chen W, Li C-H et al An advanced in situ magnetic resonance imaging and ultrasonic theranostics nanocomposite platform: crossing the blood–brain barrier and improving the suppression of glioblastoma using iron-platinum nanoparticles in nanobubbles. ACS Appl Mater Interfaces 2021;13:26759–69. 10.1021/acsami.1c04990 34076419

[42] Wang Y, Jiang L, Zhang Y et al Fibronectin-targeting and Cathepsin B-activatable theranostic nanoprobe for MR/fluorescence imaging and enhanced photodynamic therapy for triple negative breast cancer. ACS Appl Mater Interfaces 2020;12:33564–74. 10.1021/acsami.0c10397 32633941

[43] Shevtsov M, Stangl S, Nikolaev B et al Granzyme B functionalized nanoparticles targeting membrane Hsp70-positive tumors for multimodal cancer theranostics. Small 2019;15:1900205. 10.1002/smll.201900205 30828968

[44] Du Y, Liu X, Liang Q et al Optimization and design of magnetic ferrite nanoparticles with uniform tumor distribution for highly sensitive MRI/MPI performance and improved magnetic hyperthermia therapy. Nano Lett 2019;19:3618–26. 10.1021/acs.nanolett.9b00630 31074627

[45] Dehvari K, Lin P-T, Chang J-Y. Fluorescence-guided magnetic nanocarriers for enhanced tumor targeting photodynamic therapy. J Mater Chem B 2018;6:4676–86. 10.1039/C8TB00734A 32254412

[46] Wei J, Shuai X, Wang R et al Clickable and imageable multiblock polymer micelles with magnetically guided and PEG-switched targeting and release property for precise tumor theranosis. Biomaterials 2017;145:138–53. 10.1016/j.biomaterials.2017.08.005 28863308

[47] Wang Z, Qiao R, Tang N et al Active targeting theranostic iron oxide nanoparticles for MRI and magnetic resonance-guided focused ultrasound ablation of lung cancer. Biomaterials 2017;127:25–35. 10.1016/j.biomaterials.2017.02.037 28279919 PMC5400286

[48] Huang J, Qian W, Wang L et al Functionalized milk-protein-coated magnetic nanoparticles for MRI-monitored targeted therapy of pancreatic cancer. Int J Nanomedicine 2016;11:3087–99. 10.2147/IJN.S92722 27462153 PMC4939990

[49] Shi G, Liu X, Du Y et al RGD targeted magnetic ferrite nanoparticles enhance antitumor immunotherapeutic efficacy by activating STING signaling pathway. iScience 2024;27:109062. 10.1016/j.isci.2024.109062 38660408 PMC11039334

[50] Zhang D, Ye Z, Liu H et al Cell membrane coated smart two-dimensional supraparticle for *in vivo* homotypic cancer targeting and enhanced combinational theranostics. Nanotheranostics 2021;5:275–87. 10.7150/ntno.57657 33654654 PMC7914337

[51] Veroniaina H, Wu Z, Qi X. Innate tumor-targeted nanozyme overcoming tumor hypoxia for cancer theranostic use. J Adv Res 2021;33:201–13. 10.1016/j.jare.2021.02.004 34603790 PMC8463960

[52] Huang H, Fang L, Wansapura J et al Cancer-targeted pro-theranostic bi-metallic organo-coordination nanoparticles. Theranostics 2025;15:1205–20. 10.7150/thno.99863 39816680 PMC11729564

[53] Ma Q, Wu S, Yang L et al Hyaluronic acid-guided cerasome nano-agents for simultaneous imaging and treatment of advanced atherosclerosis. Adv Sci 2023;10:2202416. 10.1002/advs.202202416 PMC9929131 36529695

[54] Li B, Li Y, Chen S et al VEGF mimetic peptide-conjugated nanoparticles for magnetic resonance imaging and therapy of myocardial infarction. J Control Release 2023;360:44–56. 10.1016/j.jconrel.2023.06.017 37330014

[55] Yang L, Tang J, Yin H et al Self-assembled nanoparticles for tumor-triggered targeting dual-mode NIRF/MR imaging and photodynamic therapy applications. ACS Biomater Sci Eng 2022;8:880–92. 10.1021/acsbiomaterials.1c01418 35099181

[56] Maiseyeu A, Di L, Ravodina A et al Plaque-targeted, proteolysis-resistant, activatable and MRI-visible nano-GLP-1 receptor agonist targets smooth muscle cell differentiation in atherosclerosis. Theranostics 2022;12:2741–57. 10.7150/thno.66456 35401813 PMC8965488

[57] An R, Liu L, Wei S et al Controlling disassembly of paramagnetic prodrug and photosensitizer nanoassemblies for on-demand orthotopic glioma theranostics. ACS Nano 2022;16:20607–21. 10.1021/acsnano.2c07491 36508254

[58] Fan Z, Jiang B, Zhu Q et al Tumor-specific endogenous FeII-activated, MRI-guided self-targeting gadolinium-coordinated theranostic nanoplatforms for amplification of ROS and enhanced chemodynamic chemotherapy. ACS Appl Mater Interfaces 2020;12:14884–904. 10.1021/acsami.0c00970 32167740

[59] Wang S, Qi G, Zhang Z et al cRGD-conjugated GdIO nanoclusters for the theranostics of pancreatic cancer through the combination of T1–T2 dual-modal MRI and DTX delivery. Molecules 2023;28:6134. 10.3390/molecules28166134 37630386 PMC10459307

[60] Vazquez-Prada KX, Lam J, Kamato D et al Targeted molecular imaging of cardiovascular diseases by iron oxide nanoparticles. Arterioscler Thromb Vasc Biol 2021;41:601–13. 10.1161/ATVBAHA.120.315404 33356385

[61] Stanicki D, Vangijzegem T, Ternad I et al An update on the applications and characteristics of magnetic iron oxide nanoparticles for drug delivery. Expert Opin Drug Deliv 2022;19:321–35. 10.1080/17425247.2022.2047020 35202551

[62] Schwenk MH . Ferumoxytol: a new intravenous iron preparation for the treatment of iron deficiency Anemia in patients with chronic kidney disease. Pharmacotherapy 2010;30:70–9. 10.1592/phco.30.1.70 20030475

[63] Costa da Silva M, Breckwoldt MO, Vinchi F et al Iron induces anti-tumor activity in tumor-associated macrophages. Front Immunol 2017;8:1479. 10.3389/fimmu.2017.01479 29167669 PMC5682327

[64] Szwed M, Marczak A. Application of nanoparticles for magnetic hyperthermia for cancer treatment—the current state of knowledge. Cancers (Basel) 2024;16:1156. 10.3390/cancers16061156 38539491 PMC10969623

[65] Schleich N, Po C, Jacobs D et al Comparison of active, passive and magnetic targeting to tumors of multifunctional paclitaxel/SPIO-loaded nanoparticles for tumor imaging and therapy. J Control Release 2014;194:82–91. 10.1016/j.jconrel.2014.07.059 25178270

[66] Javid H, Oryani MA, Rezagholinejad N et al RGD peptide in cancer targeting: benefits, challenges, solutions, and possible integrin–RGD interactions. Cancer Med 2024;13:e6800. 10.1002/cam4.6800 38349028 PMC10832341

[67] de Vries IJM, Lesterhuis WJ, Barentsz JO et al Magnetic resonance tracking of dendritic cells in melanoma patients for monitoring of cellular therapy. Nat Biotechnol 2005;23:1407–13. 10.1038/nbt1154 16258544

[68] Mertens ME, Koch S, Schuster P et al USPIO-labeled textile materials for non-invasive MR imaging of tissue-engineered vascular grafts. Biomaterials 2015;39:155–63. 10.1016/j.biomaterials.2014.10.076 25465443

[69] Mertens ME, Hermann A, Bühren A et al Iron oxide-labeled collagen scaffolds for non-invasive MR imaging in tissue engineering. Adv Funct Mater 2014;24:754–62. 10.1002/adfm.201301275 24569840 PMC3837415

[70] Zhang L, Roy S, Guo B. The role of manganese-based MRI contrast agents for cancer theranostics: where do we stand in 2025? Theranostics 2025;15:4147–74. 10.7150/thno.108705 40213669 PMC11980652

[71] Lin L, Song J, Song L et al Simultaneous fenton-like Ion delivery and glutathione depletion by MnO2-based nanoagent to enhance chemodynamic therapy. Angew Chem 2018;130:4996–5000. 10.1002/ange.201712027 29488312

[72] Wang C, Guan Y, Lv M et al Manganese increases the sensitivity of the cGAS-STING pathway for double-stranded DNA and is required for the host defense against DNA viruses. Immunity 2018;48:675–687.e7. 10.1016/j.immuni.2018.03.017 29653696

[73] Pan J, Wang Y, Pan H et al Mimicking drug-substrate interaction: a smart bioinspired technology for the fabrication of theranostic nanoprobes. Adv Funct Mater 2017;27:1603440. 10.1002/adfm.201603440

[74] Zhou C, Hou S, Huang C et al A Mn-doped calcium phosphate nanoparticle-based multifunctional nanocarrier for targeted drug delivery and cellular MR imaging. J Nanopart Res 2022;24:17. 10.1007/s11051-022-05397-4

[75] Robertson AG, Rendina LM. Gadolinium theranostics for the diagnosis and treatment of cancer. Chem Soc Rev 2021;50:4231–44. 10.1039/D0CS01075H 33599224

[76] Koushki K, Shahbaz SK, Mashayekhi K et al Anti-inflammatory action of statins in cardiovascular disease: the role of inflammasome and toll-like receptor pathways. Clinic Rev Allerg Immunol 2021;60:175–99. 10.1007/s12016-020-08791-9 PMC7985098 32378144

[77] Bérard C, Desgranges S, Dumas N et al Perfluorocarbon nanodroplets as potential nanocarriers for brain delivery assisted by focused ultrasound-mediated blood-brain barrier disruption. Pharmaceutics 2022;14:1498. 10.3390/pharmaceutics14071498 35890391 PMC9323719

[78] Patel SK, Janjic JM. Macrophage targeted theranostics as personalized nanomedicine strategies for inflammatory diseases. Theranostics 2015;5:150–72. 10.7150/thno.9476 25553105 PMC4279001

[79] Janjic JM, Shao P, Zhang S et al Perfluorocarbon nanoemulsions with fluorescent, colloidal and magnetic properties. Biomaterials 2014;35:4958–68. 10.1016/j.biomaterials.2014.03.006 24674463 PMC4058911

[80] Hsiao K-H, Huang C-M, Lee Y-H. Novel rifampicin and indocyanine green co-loaded perfluorocarbon nanodroplets provide effective in vivo photo–chemo–probiotic antimicrobility against pathogen of acne *Vulgaris cutibacterium* acnes. Nanomaterials (Basel) 2020;10:1095. 10.3390/nano10061095 32492862 PMC7353360

[81] Lee Y-H, Lin Y-C. Anti-EGFR indocyanine green-mitomycin C-loaded perfluorocarbon double nanoemulsion: a novel nanostructure for targeted photochemotherapy of bladder cancer cells. Nanomaterials (Basel) 2018;8:283. 10.3390/nano8050283 29701711 PMC5977297

[82] Lee Y-H, Ma Y-T. Synthesis, characterization, and biological verification of anti-HER2 indocyanine green-doxorubicin-loaded polyethyleneimine-coated perfluorocarbon double nanoemulsions for targeted photochemotherapy of breast cancer cells. J Nanobiotechnology 2017;15:41. 10.1186/s12951-017-0274-5 28521752 PMC5437512

[83] Bibee KP, Cheng Y, Ching JK et al Rapamycin nanoparticles target defective autophagy in muscular dystrophy to enhance both strength and cardiac function. FASEB J 2014;28:2047–61. 10.1096/fj.13-237388 24500923 PMC3986846

[84] Janjic JM, Vasudeva K, Saleem M et al Low-dose NSAIDs reduce pain via macrophage targeted nanoemulsion delivery to neuroinflammation of the sciatic nerve in rat. J Neuroimmunol 2018;318:72–9. 10.1016/j.jneuroim.2018.02.010 29519721 PMC6056892

[85] Ding L, Tang S, Tang W et al Perfluorocarbon nanoemulsions enhance therapeutic siRNA delivery in the treatment of pulmonary fibrosis. Adv Sci (Weinh) 2022;9:e2103676. 10.1002/advs.202103676 34994102 PMC8922118

[86] Li Z, Shen Y, Wang Y et al Perfluorocarbon nanoemulsions for combined pulmonary siRNA treatment of lung metastatic osteosarcoma. Adv Ther (Weinh) 2019;2:1900039. 10.1002/adtp.201900039

[87] Zhang Y, Liao Y, Bai F et al Genetically engineered eukaryocyte-bacteria hybrid membrane-camouflaged nanoemulsion for three-pronged synergistic cancer therapy. Nano Today 2024;54:102076. 10.1016/j.nantod.2023.102076

[88] Vu-Quang H, Vinding MS, Nielsen T et al Theranostic tumor targeted nanoparticles combining drug delivery with dual near infrared and 19F magnetic resonance imaging modalities. Nanomedicine 2016;12:1873–84. 10.1016/j.nano.2016.04.010 27133191

[89] State of the Technology . *Focused Ultrasound Foundation*. 2024. https://www.fusfoundation.org/the-technology/state-of-the-technology/.

[90] Shin SH, Park E-J, Min C et al Tracking perfluorocarbon nanoemulsion delivery by 19F MRI for precise high intensity focused ultrasound tumor ablation. Theranostics 2017;7:562–72. 10.7150/thno.16895 28255351 PMC5327634

[91] Cyrus T, Zhang H, Allen JS et al Intramural delivery of rapamycin with αvβ3-targeted paramagnetic nanoparticles inhibits stenosis after balloon injury. Arterioscler Thromb Vasc Biol 2008;28:820–6. 10.1161/ATVBAHA.107.156281 18292395 PMC2727458

[92] Bouvain P, Thomy K, Prinz AM et al Theranostic toolbox for neutrophil functionalization. Adv Sci (Weinh) 2025;12:e04412. 10.1002/advs.202504412 40583854 PMC12442632

